# Effect of *CYP4F2* Polymorphisms on Ticagrelor Pharmacokinetics in Healthy Chinese Volunteers

**DOI:** 10.3389/fphar.2021.797278

**Published:** 2022-02-25

**Authors:** Shanshan Nie, Kaifeng Chen, Chengxian Guo, Qi Pei, Chan Zou, Liangyuan Yao, Hongbo Yuan, Xia Zhao, Ran Xie, Xu He, Jie Huang, Guoping Yang

**Affiliations:** ^1^ Center of Clinical Pharmacology, The Third Xiangya Hospital, Central South University, Changsha, China; ^2^ Department of Pharmacy, The Third Xiangya Hospital, Central South University, Changsha, China; ^3^ Hunan Qianjin Xiangjiang Pharmaceutical Co., Ltd, Zhuzhou, China; ^4^ Department of Pharmacy, Peking University First Hospital, Beijing, China

**Keywords:** ticagrelor, pharmacokinetics, CYP4F2, genetic polymorphisms, single nucleotide polymorphism

## Abstract

**Background:** Ticagrelor belongs to a new class of P2Y_12_ receptor inhibitor that has been widely used for antiplatelet therapy. This study aimed to explore the effect of single nucleotide polymorphisms (SNPs) in metabolic enzymes, transporters, and other relevant variants on the pharmacokinetics (PK) of ticagrelor and its active metabolite, AR-C124910XX.

**Methods:** The study population comprised 68 healthy Chinese volunteers who were enrolled in a ticagrelor bioequivalence clinical trial. The PK profile of ticagrelor was evaluated after orally administering a single 90-mg dose of ticagrelor in tablet form. The plasma concentrations of ticagrelor and AR-C124910XX were determined through liquid chromatography–tandem mass spectrometry. Plasma DNA samples were used to explore the effect of gene polymorphisms on the PK of ticagrelor and AR-C124910XX with whole-exome sequencing.

**Results:** Female participants had a higher maximum plasma concentration/weight ratio (*C*_max_/*W*; *p* < 0.001) and a shorter half-life (*T*_1/2_; *p* < 0.05) for ticagrelor than their male counterparts. In addition, a higher area under the curve/weight ratio (AUC/*W*; *p* < 0.001), and longer *T*_1/2_ (*p* < 0.001) and time to reach the maximum plasma concentration (*T*_max_; *p* < 0.001), as well as a lower apparent drug clearance (CL/F; *p* < 0.001), were observed among healthy volunteers in the fed trial compared to those enrolled in the fasting trial. For AR-C124910XX, higher *C*_max_/*W* (*p* < 0.001) and AUC/*W* (*p* < 0.001) but lower CL/F (*p* < 0.001) and apparent volume of distribution (*V*_d_/F; *p* < 0.001) were observed among female participants. Healthy volunteers enrolled in the fasting trial exhibited higher *C*_max_/*W* (*p* < 0.001) and AUC/*W* (*p* < 0.01), shorter *T*_max_ (*p* < 0.001), and lower CL/F (*p* < 0.001) and *V*_d_/F (*p* < 0.001) than those enrolled in the fed trial. Upon confirmation through multivariate analysis, the *CYP4F2* rs2074900 A/A carriers were associated with higher *C*_max_/*W* and AUC/*W* and lower CL/F and *V*_d_/F than the *CYP4F2* rs2074900 A/G and G/G carriers.

**Conclusion:** This study is the first to show that the *CYP4F2* rs2074900 SNP had a remarkable effect on ticagrelor PK, which is significant since it adds to the limited pharmacogenetic information on ticagrelor.

## Introduction

Cardiovascular diseases are the leading cause of mortality worldwide, and up to 30% of patients die of acute coronary events every year (Rodrigues et al., 2020). Percutaneous coronary intervention is a first-line therapy for patients with acute coronary syndrome (ACS). Guidelines of the American College of Cardiology/American Heart Association recommend the administration of dual antiplatelet therapy (aspirin + a platelet P2Y_12_ inhibitor) to prevent atherothrombotic complications for 12 months (Capodanno et al., 2018). Ticagrelor is one of the most common P2Y_12_ inhibitors, which was licensed in 2010 by AstraZeneca (Cambridge, UK). In the PLATO (PLATelet Inhibition and Patients Outcome trial) trial, ticagrelor reduced the total number of cardiovascular events, including initial and subsequent recurrent cardiovascular events (Kohli et al., 2013). In addition, the TICO (Ticagrelor Monotherapy After 3 Months in the Patients Treated With New Generation Sirolimus-Eluting Stent for Acute Coronary Syndrome) clinical trial reported that ticagrelor monotherapy after 3 months of dual antiplatelet therapy significantly reduced the incidence of composite outcomes of major bleeding and cardiovascular events at 12 months for patients with ACS treated with drug-eluting stents (Kim et al., 2019; Kim et al., 2020). The guidelines of the European Society of Cardiology have recommended the administration of ticagrelor therapy for patients at moderate to high ischemic risk with a class of recommendation Ι, level of evidence B (Rodriguez and Mahaffey, 2016).

Unlike clopidogrel, a prodrug that is converted to the active metabolite by hepatic cytochrome P450 enzyme *CYP2C19*, ticagrelor is an adenine nucleoside analogue that can reversibly target the P2Y_12_ receptor and block adenosine diphosphate (ADP)-mediated platelet aggregation (Wernly et al., 2020). In addition, it has been reported that the carriers of *CYP2C19* loss-of-function alleles are at a higher risk of ischemic events than are non-carriers for clopidogrel (Pereira et al., 2020). However, the genotype of *CYP2C19* has no effect on the pharmacokinetics (PK) and the curative effect of ticagrelor (Wallentin et al., 2010). *CYP3A4* and *CYP3A5* enzymes play a significant role in ticagrelor metabolism, but information regarding the effects of the genetic polymorphisms of metabolic enzymes and transporters on the PK of ticagrelor and its active metabolite, AR-C124910XX, is limited. Data from a clinical trial further corroborated the finding that the CYP3A4*22 allele impaired the elimination and increased the exposure of ticagrelor in healthy Finnish volunteers of Caucasian ethnicity (Holmberg et al., 2019). Furthermore, the CYP3A4*1G allele also increased the clearance of its active metabolite, AR-C124910XX, and had no effect on the PK of ticagrelor in healthy Chinese volunteers (Liu et al., 2018). Moreover, genetic variants of *SLCO1B1* rs113681054, SLCO1B1*5 (rs4149056), and CYP3A5*3 (rs776746) were also reported to have no relevance in the PK of ticagrelor in healthy Chinese volunteers (Li et al., 2017a). Platelet endothelial aggregation receptor (*PEAR1*; location: 1q23.1) plays a vital role in platelet adhesion and platelet aggregation *via* sustaining αIIbβ3 activation (Duconge et al., 2021). Prior studies have confirmed that *PEAR1* triggers PI3K/AKT/PTEN signaling, which leads to megakaryopoiesis and neoangiogenesis (Kauskot et al., 2013). Of note is that *PEAR1* polymorphisms attenuate the effect of antiplatelet drugs such as aspirin, clopidogrel, prasugrel, and ticagrelor (Würtz et al., 2014; Li et al., 2017b; Yao et al., 2018; Ansari et al., 2021). Alteration of drug efficacy associated with genetic variants may result from variability in gene-oriented PK; thus, it is essential to explore the relationship between *PEAR1* polymorphisms and ticagrelor PK.

Since genetic data on the PK profile of ticagrelor are inconsistent and incomprehensive at present, we performed whole-exome sequencing using DNA samples from 68 healthy Chinese volunteers to determine all gene variants potentially related to the PK of ticagrelor and AR-C124910XX. We expect our results to provide a more comprehensive understanding of the relationship between single nucleotide polymorphisms (SNPs) and ticagrelor PK and offer a reference for clinical individual antiplatelet therapy.

## Patients and Methods

### Study Population

The study population consisted of 68 healthy Chinese volunteers enrolled in a ticagrelor bioequivalence clinical trial. This study complied with the Good Clinical Practice guidelines and the tenets of the Declaration of Helsinki and was approved by the Institutional Review Board of The Third Xiangya Hospital of Central South University (IRB #18031). All participants provided informed consent for participation in the clinical trial and the pharmacogenetic study. The inclusion criteria were: non-smoking healthy men or women, aged 18–55 years, and having a body mass index (BMI) between 19 and 26 kg/m^2^. In addition, no clinically relevant physical abnormalities were detected in each subject, as revealed through their medical records and detailed physical examination (vital signs, laboratory analysis, and electrocardiography). The exclusion criteria were as follows: a history of drug abuse 1 year prior to the trial, having used any drugs or health products including herbs in the past 2 weeks, having participated in any clinical trial or having used any investigational drug within 3 months, having a history of blood donation or blood loss volume of >200 ml or having accepted transfusion of blood products within 3 months, being pregnant or lactating, and having consumed grapefruit in the previous 48 h.

### Study Design

The ticagrelor bioequivalence study included fasting and fed trials. Each trial was a phase I, randomized, open-label, single-center, single-dose, two-product, two-period crossover design to compare the PK bioequivalence of biosimilar drugs and its reference biopharmaceutical Brilinta®. In each period, subjects received a single 90-mg oral dose of a ticagrelor tablet, either the reference or test formulation, after overnight fasting of at least 10 h. The washout period was 7 days. Volunteers took ticagrelor on an empty stomach and fasted for another 4 h in the fasting trial. In contrast, a high-fat meal was consumed within 30 min prior to drug dosing in the fed trial. Furthermore, they were forbidden to carry out vigorous activity and ate unified lunch and dinner after 4 and 10 h of the dose.

### Sample Collection and Bioanalysis

Blood samples for PK evaluation were harvested from peripheral veins at 20 min (fasting trial) and 30 min (fed trial) before dosing and at 20 min (fasting trial)/0.5 h (fed trial), at 40 min (fasting trial), at 1 h, 80 min, 100 min, 2 h, 140 min, 160 min, and 3, 3.5, 4, and 4.5 h (fed trial), and at 5, 6, 8, 12, 24, 36, and 48 h after dosing. Approximately 4 ml blood sample was collected in heparinized vacutainer tubes and centrifuged at 2–8°C for 10 min at 1,500 × *g* within 1 h after sample collection. Plasma samples were vertically stored at −80°C until the plasma concentration of ticagrelor was measured using validated liquid chromatography–tandem mass spectrometry. Human plasma samples were precipitated with acetonitrile (ACN) using ticagrelor-D7 and AR-C124910XX-D7 as internal standards. To be more specific, 40.0 μl of standard curve samples, quality control samples, unknown samples, and blank matrix was transferred into a 96-well polypropylene plate, and 20.0 μl of internal standard working fluid (250 ng/ml in 50% ACN) was added to all samples in addition to the blank sample, in which a 50% ACN solution of 20.0 μl was added. Subsequently, 340 μl of ACN was added to all samples. Separation was carried out on a Waters ACQUITY UPLC HSS T3 (2.1×100 mm, 1.8 μm) column with a mobile phase of NH_4_Ac-ACN (including 0.1% formic acid) at a flow rate of 0.6 ml/min. Electrospray ionization (ESI) was performed in multiple reaction monitoring (MRM) negative ion mode using target ions at a mass-to-charge ratio (*m*/*z*) of 521.3→*m*/*z* 361.2 (ticagrelor) and *m*/*z* 477.3→*m*/*z* 361.2 (AR-C124910XX). The calibration curves of ticagrelor and AR-C124910XX were obtained in ranges of ∼5.00–2000.00 and ∼2.50–1,000.00 ng/ml with good linearity (*R* ^2^ > 0.99) and an intra- and inter-assay variability of <15%. The lower and upper limits of quantification were 5.00 and 2,000.00 ng/ml for ticagrelor and 2.50 and 1,000 ng/ml for AR-C124910XX, respectively. The accuracy and the intra- and inter-day precision of the different quality control samples were adequate for the method and are indicated in Supplementary Table S1.

### PK Analysis

PK parameters were calculated using a non-compartmental approach with Phoenix^®^ WinNonlin 8.2 (Cetara USA, Inc., Princeton, NJ, USA). The maximum plasma concentration (*C*_max_) and the time to reach the maximum plasma concentration (*T*_max_) were both obtained directly from the concentration–time curves. Linear trapezoidal integration was used to calculate the area under the curve (AUC) between the predose (*t* = 0 h) and the final (*t* = 48 h) time points, namely, AUC_0–*t*_. The remaining AUC from 48 h to infinity (AUC_*t*–∞_) was obtained by dividing the last measured concentration (*C*_*t*_) by the constant of elimination (*k*_e_). The terminal rate constant (*k*_e_) was calculated by linear regression of the log-linear part of the concentration–time curve. The AUC between 0 and ∞ (AUC_0–∞_) was calculated as AUC_0–*t*_ + AUC_*t*–∞_. Apparent drug clearance (CL/F) was calculated as the dose divided by the AUC_0–∞_ and weight (*W*). Similarly, the apparent volume of distribution (*V*_d_/F) was calculated as CL/F divided by *k*_e_. Half-life (*T*_1/2_) was estimated to be 0.693/*k*_e_.

### DNA Extraction and Whole-Exome Sequencing

Genomic DNA was extracted using a Wizard® Genomic DNA Purification Kit (Promega, Madison, WI, USA) in accordance with the manufacturer’s instructions. In total, qualified genomic DNA (3 μg) from each sample was considered for initial whole-exome sequencing, from which the genomic regions of interest were captured using the Agilent SureSelectXT Target Enrichment Kit for Illumina multiplex sequencing at Capital Bio Technology Co., Ltd (Beijing, China). Ultimately, a total of 35 variants in 10 genes were included on the basis of the identified SNPs of metabolic enzymes (CYP/UGT enzymes), transporters (*ABCB1*or *SLCO1B1*), and other relevant variants (*PEAR1*). The included variants were the following: *CYP4F2* rs2074900; *CYP2C9* rs17847029, rs1057911, and rs9332245; *CYP2B6* rs2279342, rs35930845, rs3745276, rs434606, and rs56156262; CYP2C19*2 (rs4244285), rs12769205, and rs3758580; CYP3A4*18 (rs28371759); *CYP3A5* rs15524, rs4646453, and rs6977165; *UGT2B7* rs141257984, rs28365062, rs7438284, rs5013211, and rs4257713; *PEAR1* rs77235035, rs735953, and rs189160314; *SLCO1B1* rs4149032, rs4149034, rs2306283, rs4149057, rs2291075, and rs2291076; and *ABCB1* rs2235048, rs2235047, rs1045642, rs2235013, and rs2235033.

### Statistical Analysis

R (version 4.0.2) was used for statistical analysis. The variables AUC and *C*_max_ were divided by the weight (AUC/*W* and *C*_max_/*W*, respectively) to correct the impact of weight. All PK parameters were log-transformed to normalize distributions. Initially, a univariate analysis was performed, where the mean of the PK parameters was compared in accordance with categorical variables (e.g., sex, food effect, and SNPs). A *t*-test (two groups) or an analysis of variance (ANOVA) followed by *post-hoc* Bonferroni correction (more than two groups) was conducted to evaluate the associations between the categorical variables and PK parameters. A *p*-value <0.05 was considered significant. Afterward, multivariate analysis was performed using the multiple linear regression model. As dependent variables, all PK parameters were analyzed; as independent variables, only variables with *p* < 0.05 in the univariate analysis were included. Bonferroni correction for multiple comparisons was performed. The significant *p*-value of <0.05 was divided by the number of variables introduced in multivariate analysis. All genotyped variants were analyzed using the chi-square test with the HardyWeinberg package to confirm whether the gene distribution complied with the Hardy–Weinberg equilibrium.

## Results

### Demographic Characteristics

The study population (*N* = 68) comprised 45 (66%) men and 23 (34%) women. Women had significantly lower weight and height (*p* < 0.0001) compared with men, with similar age and BMI (Table 1). In addition, the age, weight, height, and BMI were comparable between the fasting and fed trials.

**TABLE 1:** Demographic characteristics of the healthy volunteers in this study

	***N***	**Age (years)**	**SD**	**BMI (kg/m** ^**2**^ **)**	**SD**	**Weight (kg)**	**SD**	**Height (cm)**	**SD**
Sex									
Male	45	23.87	4.56	22.13	1.58	62.32*	6.04	167.74*	6.05
Female	23	22.35	4.12	21.96	1.88	54.63	4.80	157.78	4.08
Food									
Fed	34	24.18	5.02	21.95	1.40	58.81	6.04	163.57	6.67
Fasting	34	22.53	3.51	22.20	1.88	60.98	7.30	165.29	7.53
Total	68	23.36	4.27	22.08	1.64	59.90	6.67	164.43	7.10

*BMI*, body mass index; *SD*, standard deviation

*p < 0.05 after *t*-test

### Pharmacokinetics

For ticagrelor, a significantly higher *C*_max_/*W* (*p* < 0.001) and a shorter *T*_1/2_ (*p* < 0.05) were observed among women than among men (Table 2). In addition, healthy volunteers enrolled in the fed trial had higher values of AUC/*W* (*p* < 0.001), *T*_1/2_ (*p* < 0.001), and *T*_max_ (*p* < 0.001), as well as lower CL/F (*p* < 0.001), than those enrolled in the fasting trial. For AR-C124910XX, the *C*_max_/*W* (*p* < 0.001) and AUC/*W* (*p* < 0.001) values were higher and the CL/F (*p* < 0.001) and *V*_d_/F (*p* < 0.001) values were lower in women than in men. Moreover, higher *C*_max_/*W* (*p* < 0.001) and AUC/*W* (*p* < 0.01) and a shorter *T*_max_ (*p* < 0.001) and lower CL/F (*p* < 0.001) and *V*_d_/F (*p* < 0.001) were observed in healthy volunteers enrolled in the fasting trial than those enrolled in the fed trial.

**TABLE 2:** Main pharmacokinetic parameters of ticagrelor and AR-C124910XX based on sex and food

	***C***_**max**_ /*W* (ng/kg*ml)	**SD**	***T***_**max**_ **(h)**	**SD**	**AUC**_**0–*t***_ /W (ng*h/kg*ml)	**SD**	**AUC**_**0–∞**_ /W (ng*h/kg*ml)	**SD**	CL/F (L/h*kg)	**SD**	***V***_**d**_ **/F (L/kg)**	**SD**	***T***_**1/2**_ **(h)**	**SD**
Ticagrelor														
Sex														
Male	9.29	2.76	2.45	1.14	69.00	24.60	70.20	25.10	0.37	0.11	3.60	0.76	7.05	1.16
Female	13.20*	4.86	2.39	1.03	82.00	27.50	83.00	27.60	0.40	0.12	3.63	0.76	6.43*	0.85
Food														
Fed	9.80	3.48	2.89*	1.19	83.50*	26.30	84.80*	26.80	0.33*	0.09	3.45	0.67	7.33*	1.09
Fasting	11.40	4.40	1.97	0.77	63.20	22.00	64.20	21.90	0.42	0.12	3.78	0.82	6.34	0.88
AR-C124910XX														
Sex														
Male	2.50	1.05	3.12	1.02	25.50	7.45	26.50	7.56	0.94	0.22	12.40	4.16	9.08	1.66
Female	4.22*	1.92	3.18	1.06	44.80*	15.10	46.30*	15.80	0.71*	0.20	9.00*	2.60	8.87	1.62
Food														
Fed	2.08	0.76	3.66	1.07	27.70	10.10	28.80	10.20	0.99	0.22	13.50	4.44	9.28	1.62
Fasting	4.08*	1.62	2.63*	0.68	36.40*	16.10	37.50*	16.80	0.74*	0.20	9.08*	1.86	8.73	1.63

*C*_*max*_ */W*, maximum plasma concentration/weight ratio; *SD*, standard deviation; *T*_*max*_, time to reach maximum plasma concentration; *AUC*_*0–t*_ */W*, area under the curve between the predose and final time points/weight; *AUC*_*0–∞*_ */W*, AUC between the predose and infinity/weight; *CL/F*, apparent drug clearance; *V*_*d*_ */F*, apparent volume of distribution; *T*_*1/2*_, half-life

*p < 0.05 after *t*-test

All included SNPs were in Hardy–Weinberg equilibrium. Genetic polymorphism was associated with ticagrelor pharmacokinetic variability. Table 3 shows the associations between SNPs and ticagrelor PK with statistical significance. On univariate analysis, the *C*_max_/*W* (*p* < 0.05) and AUC/*W* (*p* < 0.05) were higher and the CL/F (*p* < 0.05) and *V*_d_/F (*p* < 0.05) were lower among *CYP4F2* rs2074900 A/A carriers after *post-hoc* Bonferroni correction than in *CYP4F2* rs2074900 A/G and G/G carriers. On multivariate analysis, since sex and food intake were significantly related to PK variability, they were included to rule out the impact of confounding factors. The level of significance was set at *p* = 0.0167 (*p* < 0.05 divided by 3, the number of variables introduced in the multivariate analysis). After confirmation through multivariate analysis, the *C*_max_/*W* and AUC/*W* values were lower and the CL/F and *V*_d_/F values were higher among *CYP4F2* rs2074900 A/G and G/G carriers than among *CYP4F2* rs2074900 A/A carriers (unstandardized *β* coefficients = −0.182, −0.168, 0.150, and 0.115; *p* < 0.01 for all; *R* ^2^ = 0.27, 0.296, 0.269, and 0.139). Moreover, the AUC/*W* values were lower among *PEAR1* rs77235035 A/A carriers than among A/C carriers (*P*_ANOVA_ < 0.05), but not among C/C carriers; hence, this SNP was excluded from multivariate analysis (Table 3). Regarding AR-C124910X, no associations were observed between SNPs and their PK parameters. Non-significant associations between SNPs and ticagrelor/AR-C124910XX PK are shown in Tables 4 and 5.

**TABLE 3:** Ticagrelor pharmacokinetic parameters based on genotypes with statistical significance

		***N***	***C***_**max**_ /*W* (ng/kg*ml)	**SD**	***T***_**max**_ **(h)**	**SD**	**AUC**_**0–*t***_ /*W* (ng*h/kg*ml)	**SD**	**AUC**_**0–∞**_ /*W* (ng*h/kg*ml)	**SD**	CL/F (L/h*kg)	**SD**	***V***_**d**_ **/F (L/kg)**	**SD**	***T***_**1/2**_ **(h)**	**SD**
*CYP4F2* rs2074900	A/A	5	15.50*	5.34	2.43	1.19	109.00*	38.10	110.00*	38.40	0.27*	0.06	2.79*	0.53	7.38	0.83
	A/G	22	10.70	4.76	2.58	1.16	71.00	21.90	72.10	21.90	0.41	0.14	3.86	1.00	6.76	0.84
	G/G	41	9.95	2.97	2.35	1.08	70.30	24.00	71.40	24.50	0.38	0.09	3.58	0.54	6.81	1.25
*PEAR1* rs77235035	A/A	7	9.82	2.61	2.38	1.51	53.50*	18.90	54.20*	18.70	0.47	0.19	3.92	1.32	5.92	0.66
	A/C	36	10.90	4.46	2.38	1.02	78.00	28.90	79.20	29.20	0.37	0.10	3.55	0.66	6.92	1.14
	C/C	25	10.40	3.74	2.51	1.13	72.20	21.30	73.50	21.60	0.37	0.10	3.62	0.70	6.97	1.06

*C*_*max*_ */W*, maximum plasma concentration/weight ratio; *SD*, standard deviation; *T*_*max*_, time to reach maximum plasma concentration; *AUC*_*0–t*_ */W*, area under the curve between the predose and final time points/weight; *AUC*_*0–∞*_ */W*, AUC between the predose and infinity/weight; *CL/F*, apparent drug clearance; *V*_*d*_ */F*, apparent volume of distribution; *T*_*1/2*_, half-life

*p* < 0.05 after multivariate analysis (linear regression, which included the following variables: sex, food effect, and *CYP4F2* rs2074900; *PEAR1* rs77235035 was excluded from the analysis). Bold values: *p* < 0.0167 after multivariate analysis (Bonferroni correction for multiple testing significance threshold)

*p < 0.05 after ANOVA and Bonferroni *post-hoc* (for *CYP4F2* rs2074900: A/A, *vs*. A/G, and G/G; for *PEAR1* rs77235035: A/A, *vs*. A/C)

**TABLE 4:** Ticagrelor pharmacokinetic parameters based on genotypes without statistical significance

		***N***	***C***_**max**_ /*W* (ng/kg*ml)	**SD**	***T***_**max**_ **(h)**	**SD**	**AUC**_**0–*t***_ /*W* (ng*h/kg*ml)	**SD**	**AUC**_**0–∞**_ /*W* (ng*h/kg*ml)	**SD**	CL/F (L/h*kg)	**SD**	***V***_**d**_ **/F (L/kg)**	**SD**	***T***_**1/2**_ **(h)**	**SD**
*CYP2C9* rs17847029	C/C	58	10.40	3.90	2.47	1.18	72.40	27.00	73.60	27.30	0.38	0.12	3.61	0.80	6.89	1.14
	T/C	10	11.90	4.68	2.20	0.39	79.00	20.90	79.70	21.00	0.38	0.05	3.61	0.52	6.55	0.80
*CYP2C9* rs1057911	A/A	60	10.70	4.16	2.42	1.08	73.40	26.40	74.50	26.70	0.38	0.12	3.62	0.77	6.85	1.08
	T/A	8	9.60	2.71	2.48	1.29	72.80	26.30	74.30	26.60	0.37	0.11	3.52	0.72	6.77	1.32
*CYP2C9* rs9332245	A/T	8	9.60	2.71	2.48	1.29	72.80	26.30	74.30	26.60	0.37	0.11	3.52	0.72	6.77	1.32
	T/T	60	10.70	4.16	2.42	1.08	73.40	26.40	74.50	26.70	0.38	0.12	3.62	0.77	6.85	1.08
*CYP2B6* rs2279342	A/A	40	10.10	3.98	2.43	1.13	72.70	29.30	73.80	29.70	0.38	0.13	3.65	0.90	6.85	1.07
	T/A	24	11.20	4.26	2.56	1.10	74.30	22.40	75.30	22.60	0.37	0.08	3.49	0.42	6.71	1.02
	T/T	4	12.40	2.32	1.58	0.17	74.90	15.30	76.60	15.40	0.40	0.16	4.02	0.85	7.43	1.89
*CYP2B6* rs35930845	C/C	34	11.50	3.87	2.31	1.04	76.80	26.20	78.00	26.60	0.37	0.10	3.50	0.64	6.83	1.19
	G/C	30	9.75	3.87	2.41	1.11	69.50	26.30	70.50	26.40	0.39	0.13	3.68	0.85	6.78	1.04
	G/G	4	9.03	5.55	3.50	1.22	73.70	27.80	74.80	27.70	0.39	0.12	4.02	0.95	7.33	0.84
*CYP2B6* rs3745276	A/A	7	11.30	5.21	2.52	1.37	75.40	15.70	76.30	16.00	0.35	0.05	3.24	0.27	6.50	0.64
	A/G	32	10.70	3.92	2.31	1.04	77.00	33.10	78.20	33.50	0.38	0.13	3.55	0.88	6.83	1.24
	G/G	29	10.30	3.95	2.53	1.13	68.90	18.30	70.00	18.40	0.39	0.10	3.77	0.66	6.93	1.03
*CYP2B6* rs434606	A/A	12	11.70	3.65	2.03	0.72	70.60	16.70	71.80	16.70	0.40	0.10	3.86	0.61	6.96	1.18
	A/G	38	10.00	3.97	2.64	1.14	69.40	25.50	70.40	25.60	0.39	0.10	3.64	0.70	6.74	1.02
	G/G	18	11.10	4.34	2.24	1.17	83.50	30.80	84.90	31.40	0.35	0.14	3.38	0.93	6.96	1.26
*CYP2B6* rs56156262	A/A	13	12.50	4.23	2.57	1.20	89.00	33.60	90.30	34.10	0.34	0.10	3.27	0.50	6.92	1.12
	A/G	40	10.50	4.27	2.40	1.16	70.50	25.40	71.60	25.60	0.40	0.13	3.71	0.88	6.74	1.10
	G/G	15	9.20	2.43	2.37	0.87	67.60	14.80	68.70	15.10	0.37	0.06	3.64	0.50	7.02	1.14
*CYP2C19* rs12769205	A/A	30	10.10	3.16	2.62	1.22	72.70	25.30	74.00	25.70	0.38	0.14	3.74	0.97	7.03	1.18
	G/A	31	11.10	4.89	2.24	0.98	74.40	28.50	75.40	28.80	0.37	0.09	3.52	0.57	6.83	1.01
	G/G	7	10.70	3.11	2.45	1.05	72.00	21.60	72.90	21.50	0.40	0.07	3.47	0.41	6.06	0.87
*CYP2C19* rs4244285	A/A	7	10.70	3.11	2.45	1.05	72.00	21.60	72.90	21.50	0.40	0.07	3.47	0.41	6.06	0.87
	A/G	31	11.10	4.89	2.24	0.98	74.40	28.50	75.40	28.80	0.37	0.09	3.52	0.57	6.83	1.01
	G/G	30	10.10	3.16	2.62	1.22	72.70	25.30	74.00	25.70	0.38	0.14	3.74	0.97	7.03	1.18
*CYP2C19* rs3758580	C/C	30	10.10	3.16	2.62	1.22	72.70	25.30	74.00	25.70	0.38	0.14	3.74	0.97	7.03	1.18
	T/C	31	11.10	4.89	2.24	0.98	74.40	28.50	75.40	28.80	0.37	0.09	3.52	0.57	6.83	1.01
	T/T	7	10.70	3.11	2.45	1.05	72.00	21.60	72.90	21.50	0.40	0.07	3.47	0.41	6.06	0.87
*CYP3A4* rs28371759	A/A	65	10.70	4.09	2.40	1.09	73.40	26.30	74.50	26.60	0.38	0.12	3.64	0.76	6.85	1.12
	G/A	3	9.14	1.84	2.95	1.44	73.50	28.00	74.40	28.50	0.33	0.08	3.10	0.58	6.60	0.47
*CYP3A5* rs15524	A/A	35	10.30	3.91	2.35	1.11	70.50	24.90	71.70	25.20	0.39	0.11	3.68	0.71	6.79	1.16
	G/A	25	10.90	4.01	2.33	0.94	78.70	29.00	79.70	29.40	0.36	0.13	3.47	0.84	6.87	1.18
	G/G	8	10.70	4.95	3.06	1.42	69.50	22.20	70.50	22.40	0.38	0.08	3.78	0.69	6.97	0.50
*CYP3A5* rs4646453	A/A	6	10.20	5.50	3.00	1.47	65.70	22.80	66.70	23.00	0.38	0.09	3.75	0.80	6.86	0.53
	A/C	25	11.10	4.08	2.41	1.03	79.50	29.20	80.50	29.60	0.37	0.13	3.51	0.84	6.85	1.14
	C/C	37	10.30	3.81	2.34	1.09	70.50	24.30	71.70	24.50	0.39	0.11	3.66	0.70	6.83	1.16
*CYP3A5* rs6977165	C/T	2	12.50	3.25	3.58	1.29	80.10	23.70	81.40	23.90	0.37	0.04	3.70	0.07	6.98	0.62
	T/T	66	10.50	4.05	2.39	1.09	73.20	26.40	74.30	26.70	0.38	0.12	3.61	0.77	6.83	1.11
*UGT2B7* rs141257984	G/T	5	9.77	2.25	2.00	0.41	66.10	12.90	66.90	12.80	0.39	0.08	3.80	0.45	6.88	0.87
	T/T	63	10.70	4.13	2.46	1.13	74.00	26.90	75.10	27.20	0.38	0.12	3.60	0.78	6.83	1.12
*UGT2B7* rs28365062	A/A	66	10.60	4.07	2.39	1.07	73.50	26.20	74.70	26.50	0.38	0.11	3.62	0.77	6.84	1.10
	G/A	2	9.05	0.30	3.66	1.89	68.50	32.60	69.50	33.40	0.37	0.10	3.46	0.22	6.76	1.48
*UGT2B7* rs7438284	A/A	10	8.67	2.62	2.47	1.14	64.20	16.30	65.20	16.30	0.40	0.11	3.58	0.70	6.47	1.23
	A/T	29	10.30	3.92	2.21	0.92	69.70	27.40	70.90	27.70	0.40	0.13	3.79	0.81	6.88	1.09
	T/T	29	11.50	4.33	2.63	1.24	80.20	26.60	81.30	27.00	0.35	0.09	3.45	0.70	6.92	1.08
*UGT2B7* rs5013211	A/A	30	11.60	4.25	2.58	1.26	78.80	27.30	79.90	27.60	0.36	0.10	3.48	0.71	6.86	1.11
	G/A	28	10.30	3.98	2.25	0.90	70.80	27.20	72.00	27.50	0.39	0.13	3.77	0.82	6.94	1.06
	G/G	10	8.67	2.62	2.47	1.14	64.20	16.30	65.20	16.30	0.40	0.11	3.58	0.70	6.47	1.23
*UGT2B7* rs4257713	A/A	5	8.50	0.84	2.44	1.23	70.10	13.00	71.20	12.70	0.35	0.10	3.35	0.34	6.89	1.41
	A/G	26	10.30	4.03	2.28	0.96	68.50	26.20	69.60	26.50	0.39	0.10	3.70	0.69	6.73	0.98
	G/G	37	11.10	4.22	2.53	1.19	77.20	27.30	78.40	27.60	0.37	0.12	3.59	0.84	6.91	1.17
*PEAR1* rs735953	C/C	8	10.40	5.01	2.54	1.28	62.80	28.60	63.60	28.50	0.44	0.18	3.93	1.29	6.27	0.68
	C/T	33	10.90	4.19	2.36	1.09	76.50	29.50	77.60	29.90	0.37	0.11	3.55	0.66	6.89	1.22
	T/T	27	10.30	3.61	2.48	1.09	72.70	20.60	73.90	20.80	0.37	0.09	3.60	0.68	6.94	1.02
*PEAR1* rs189160314	A/A	66	10.60	4.05	2.45	1.11	74.00	26.20	75.20	26.60	0.37	0.11	3.59	0.74	6.87	1.10
	G/A	2	11.00	3.78	1.67	0.00	51.00	3.78	52.20	4.98	0.52	0.14	4.30	1.31	5.68	0.21
*SLCO1B1* rs4149032	C/C	15	10.50	4.79	2.32	0.76	78.10	35.40	79.40	36.00	0.41	0.18	3.72	1.14	6.66	1.30
	C/T	37	10.50	3.36	2.55	1.21	73.00	24.50	74.10	24.70	0.37	0.09	3.64	0.66	6.96	1.04
	T/T	16	11.00	4.84	2.25	1.12	69.80	20.30	70.80	20.40	0.37	0.10	3.44	0.51	6.72	1.08
*SLCO1B1* rs4149034	A/A	15	10.90	5.00	2.33	1.11	71.90	19.10	72.90	19.20	0.35	0.08	3.38	0.45	6.82	1.04
	A/G	37	10.50	3.37	2.50	1.24	72.30	25.10	73.40	25.30	0.38	0.09	3.66	0.68	6.92	1.07
	G/G	16	10.50	4.63	2.34	0.74	77.20	34.40	78.50	34.90	0.41	0.17	3.73	1.10	6.66	1.26
*SLCO1B1* rs2306283	A/A	3	8.53	2.87	2.33	0.88	59.40	28.50	60.20	28.20	0.57	0.25	4.34	2.04	5.30	0.45
	A/G	31	10.80	4.15	2.53	1.10	77.80	28.80	78.90	29.00	0.38	0.10	3.68	0.76	6.87	0.84
	G/G	34	10.60	4.03	2.34	1.14	70.60	23.30	71.70	23.70	0.36	0.10	3.49	0.57	6.95	1.26
*SLCO1B1* rs4149057	C/C	4	7.77	2.79	2.50	0.80	59.00	23.30	60.10	23.00	0.51	0.24	4.25	1.67	6.10	1.65
	C/T	29	10.80	4.10	2.55	1.12	78.40	29.50	79.50	29.80	0.38	0.10	3.69	0.78	6.81	0.81
	T/T	35	10.70	4.03	2.32	1.13	70.90	23.00	72.00	23.40	0.36	0.10	3.48	0.56	6.94	1.24
*SLCO1B1* rs2291075	C/C	19	11.00	4.52	2.37	1.06	70.10	29.20	71.10	29.10	0.42	0.14	3.83	0.96	6.51	0.98
	T/C	31	10.50	3.90	2.59	1.12	76.30	25.30	77.40	25.80	0.36	0.09	3.58	0.67	7.03	0.94
	T/T	18	10.40	3.87	2.21	1.13	71.80	25.20	73.10	25.60	0.36	0.12	3.43	0.64	6.86	1.41
*SLCO1B1* rs2291076	C/C	35	10.70	4.03	2.32	1.13	70.90	23.00	72.00	23.40	0.36	0.10	3.48	0.56	6.94	1.24
	T/C	28	10.80	4.18	2.58	1.13	78.50	30.00	79.60	30.30	0.38	0.10	3.68	0.79	6.81	0.83
	T/T	5	8.37	2.76	2.33	0.78	62.30	21.50	63.40	21.20	0.48	0.21	4.15	1.47	6.24	1.46
*ABCB1* rs2235048	A/A	22	9.86	3.21	2.29	0.97	68.80	18.60	69.90	18.60	0.36	0.09	3.52	0.64	6.95	0.87
	G/A	35	10.80	4.46	2.63	1.17	75.60	29.40	76.80	29.80	0.39	0.12	3.76	0.82	6.87	1.19
	G/G	11	11.40	4.12	2.06	1.07	75.40	29.10	76.50	29.60	0.38	0.13	3.34	0.73	6.51	1.27
*ABCB1* rs2235047	A/A	23	11.00	4.14	2.25	0.96	73.00	23.70	74.00	23.90	0.39	0.11	3.61	0.79	6.69	1.02
	C/A	37	10.60	4.02	2.68	1.20	75.00	28.90	76.30	29.30	0.38	0.12	3.64	0.81	6.90	1.19
	C/C	8	9.47	3.94	1.79	0.56	67.10	20.40	67.90	20.50	0.35	0.04	3.48	0.40	6.96	0.97
*ABCB1* rs1045642	A/A	11	11.40	4.12	2.06	1.07	75.40	29.10	76.50	29.60	0.38	0.13	3.34	0.73	6.51	1.27
	A/G	35	10.80	4.46	2.63	1.17	75.60	29.40	76.80	29.80	0.39	0.12	3.76	0.82	6.87	1.19
	G/G	22	9.86	3.21	2.29	0.97	68.80	18.60	69.90	18.60	0.36	0.09	3.52	0.64	6.95	0.87
*ABCB1* rs2235013	C/C	34	10.60	4.44	2.38	1.06	72.50	23.80	73.60	24.00	0.39	0.13	3.64	0.89	6.73	1.16
	T/C	28	10.70	3.61	2.36	1.16	74.90	30.80	76.20	31.20	0.37	0.10	3.57	0.66	6.88	1.11
	T/T	6	9.99	3.96	2.97	1.11	70.90	16.50	71.90	16.40	0.35	0.03	3.64	0.37	7.21	0.75
*ABCB1* rs2235033	A/A	34	10.60	4.44	2.38	1.06	72.50	23.80	73.60	24.00	0.39	0.13	3.64	0.89	6.73	1.16
	G/A	28	10.70	3.61	2.36	1.16	74.90	30.80	76.20	31.20	0.37	0.10	3.57	0.66	6.88	1.11
	G/G	6	9.99	3.96	2.97	1.11	70.90	16.50	71.90	16.40	0.35	0.03	3.64	0.37	7.21	0.75

*C*_*max*_ */W*, maximum plasma concentration/weight ratio; *SD*, standard deviation; *T*_*max*_, time to reach maximum plasma concentration; *AUC*_*0–t*_ */W*, area under the curve between the predose and final time points/weight; *AUC*_*0–∞t*_ */W*, AUC between the predose and infinity/weight; *CL/F*, apparent drug clearance; *V*_*d*_ */F*, apparent volume of distribution; *T*_*1/2*_, half-life

**TABLE 5:** AR-C124910XX pharmacokinetic parameters based on genotypes without statistical significance

		***N***	***C***_**max**_ /*W* (ng/kg*ml)	**SD**	***T***_**max**_ **(h)**	**SD**	**AUC**_**0–*t***_ /*W* (ng*h/kg*ml)	**SD**	**AUC**_**0–∞**_ /*W* (ng*h/kg*ml)	**SD**	CL/F (L/h*kg)	**SD**	***V***_**d**_ **/F (L/kg)**	**SD**	***T***_**1/2**_ **(h)**	**SD**
*CYP4F2* rs2074900	A/A	5	3.61	2.57	3.40	1.03	37.00	23.40	38.40	24.70	0.86	0.31	11.10	4.63	9.05	1.55
	A/G	22	3.18	1.87	3.12	1.09	32.20	13.60	33.30	14.00	0.90	0.26	11.60	4.31	8.90	1.40
	G/G	41	2.96	1.34	3.12	1.01	31.30	13.20	32.50	13.50	0.85	0.22	11.10	3.91	9.06	1.79
*CYP2C9* rs17847029	C/C	58	2.95	1.50	3.19	1.06	30.50	11.60	31.60	12.00	0.87	0.24	11.50	4.15	9.11	1.65
	T/C	10	3.86	2.06	2.88	0.79	41.10	22.40	42.40	23.20	0.83	0.26	9.87	3.11	8.41	1.51
*CYP2C9* rs1057911	A/A	60	3.16	1.63	3.11	0.98	32.70	14.50	33.90	15.00	0.85	0.25	11.20	4.13	9.12	1.61
	T/A	8	2.51	1.43	3.36	1.42	26.80	8.55	27.50	8.50	0.97	0.16	11.50	3.42	8.18	1.72
*CYP2C9* rs9332245	A/T	8	2.51	1.43	3.36	1.42	26.80	8.55	27.50	8.50	0.97	0.16	11.50	3.42	8.18	1.72
	T/T	60	3.16	1.63	3.11	0.98	32.70	14.50	33.90	15.00	0.85	0.25	11.20	4.13	9.12	1.61
*CYP2B6* rs2279342	A/A	40	2.83	1.32	3.18	0.97	30.30	11.50	31.40	11.90	0.88	0.25	11.40	4.07	8.94	1.68
	T/A	24	3.41	1.97	3.16	1.18	34.90	18.00	36.20	18.50	0.83	0.24	11.30	4.22	9.32	1.56
	T/T	4	3.62	1.78	2.62	0.64	32.30	8.95	32.80	8.91	0.91	0.17	10.20	3.09	7.77	1.31
*CYP2B6* rs35930845	C/C	34	3.39	1.65	3.06	1.07	34.30	14.60	35.50	15.20	0.82	0.18	10.50	2.70	8.96	1.41
	G/C	30	2.87	1.56	3.16	1.00	30.10	12.60	31.20	13.10	0.89	0.26	11.50	4.49	8.98	1.83
	G/G	4	1.98	1.18	3.75	0.87	27.70	19.10	28.80	18.90	1.12	0.39	16.00	7.17	9.55	2.29
*CYP2B6* rs3745276	A/A	7	3.25	1.56	3.50	1.12	31.80	9.54	32.90	9.87	0.83	0.19	10.80	2.27	9.05	1.14
	A/G	32	3.01	1.62	3.08	1.06	32.20	16.20	33.50	16.80	0.87	0.22	11.40	3.35	9.11	1.47
	G/G	29	3.11	1.66	3.12	0.99	31.90	12.70	32.90	12.90	0.87	0.28	11.30	5.03	8.89	1.93
*CYP2B6* rs434606	A/A	12	3.80	1.69	2.75	0.63	36.10	12.80	37.30	13.60	0.79	0.22	9.65	2.35	8.74	1.88
	A/G	38	2.91	1.61	3.22	1.12	30.40	14.60	31.60	15.00	0.89	0.25	11.90	4.51	9.25	1.63
	G/G	18	2.95	1.51	3.24	1.02	32.70	13.70	33.70	14.00	0.87	0.23	10.90	3.65	8.66	1.48
*CYP2B6* rs56156262	A/A	13	3.12	1.12	3.32	1.09	33.60	10.30	34.90	10.40	0.86	0.23	11.60	4.92	9.12	1.71
	A/G	40	3.21	1.79	3.00	1.01	31.80	14.40	32.80	14.90	0.87	0.24	10.90	3.81	8.72	1.70
	G/G	15	2.71	1.50	3.37	1.01	31.30	16.50	32.60	17.00	0.86	0.26	12.00	3.95	9.68	1.22
*CYP2C19* rs12769205	A/A	30	2.86	1.57	3.34	1.08	30.20	11.10	31.40	11.50	0.89	0.27	12.00	4.27	9.30	1.57
	G/A	31	3.19	1.71	2.96	0.98	31.90	14.80	33.10	15.30	0.86	0.21	11.20	3.96	8.99	1.68
	G/G	7	3.52	1.37	3.10	0.96	40.40	20.30	41.40	20.80	0.77	0.21	8.53	2.00	7.87	1.40
*CYP2C19* rs4244285	A/A	7	3.52	1.37	3.10	0.96	40.40	20.30	41.40	20.80	0.77	0.21	8.53	2.00	7.87	1.40
	A/G	31	3.19	1.71	2.96	0.98	31.90	14.80	33.10	15.30	0.86	0.21	11.20	3.96	8.99	1.68
	G/G	30	2.86	1.57	3.34	1.08	30.20	11.10	31.40	11.50	0.89	0.27	12.00	4.27	9.30	1.57
*CYP2C19* rs3758580	C/C	30	2.86	1.57	3.34	1.08	30.20	11.10	31.40	11.50	0.89	0.27	12.00	4.27	9.30	1.57
	T/C	31	3.19	1.71	2.96	0.98	31.90	14.80	33.10	15.30	0.86	0.21	11.20	3.96	8.99	1.68
	T/T	7	3.52	1.37	3.10	0.96	40.40	20.30	41.40	20.80	0.77	0.21	8.53	2.00	7.87	1.40
*CYP3A4* rs28371759	A/A	65	3.12	1.63	3.11	1.03	31.90	14.30	33.10	14.80	0.88	0.24	11.40	4.06	9.02	1.62
	G/A	3	2.24	0.58	3.83	0.76	33.90	5.84	35.00	5.65	0.66	0.05	8.30	2.26	8.82	2.46
*CYP3A5* rs15524	A/A	35	3.14	1.69	3.09	1.01	31.70	13.10	32.90	13.60	0.86	0.23	11.20	4.29	9.02	1.86
	G/A	25	3.03	1.62	3.11	1.04	32.50	16.40	33.70	16.90	0.89	0.24	11.40	3.67	8.95	1.49
	G/G	8	2.96	1.37	3.50	1.14	31.70	11.40	32.80	11.50	0.85	0.32	11.20	4.47	9.14	1.07
*CYP3A5* rs4646453	A/A	6	2.98	1.56	3.44	1.04	30.70	11.80	31.80	12.10	0.85	0.37	11.20	5.01	9.28	1.07
	A/C	25	3.05	1.61	3.18	1.10	33.10	16.50	34.30	16.90	0.89	0.24	11.50	3.73	8.93	1.51
	C/C	37	3.11	1.66	3.07	1.00	31.50	12.80	32.60	13.30	0.85	0.22	11.10	4.18	9.01	1.81
*CYP3A5* rs6977165	C/T	2	3.50	0.12	4.25	1.06	39.10	7.44	40.00	7.25	0.74	0.01	9.07	1.05	8.53	1.06
	T/T	66	3.07	1.63	3.11	1.02	31.80	14.10	33.00	14.60	0.87	0.24	11.30	4.08	9.02	1.65
*UGT2B7* rs141257984	G/T	5	2.63	0.85	2.97	1.19	28.10	10.10	29.90	10.70	0.89	0.09	13.00	2.38	10.10	0.96
	T/T	63	3.12	1.65	3.16	1.02	32.30	14.30	33.40	14.80	0.86	0.25	11.10	4.12	8.92	1.65
*UGT2B7* rs28365062	A/A	66	3.11	1.62	3.13	1.01	32.40	14.10	33.60	14.50	0.86	0.24	11.20	4.05	9.03	1.65
	G/A	2	1.98	0.73	3.66	1.89	20.20	3.01	20.70	3.41	1.14	0.06	13.80	2.98	8.31	1.39
*UGT2B7* rs7438284	A/A	10	2.79	1.57	3.48	1.08	29.20	12.50	30.10	12.90	0.88	0.22	11.20	3.80	8.74	1.27
	A/T	29	3.04	1.36	2.84	0.86	31.30	11.10	32.20	11.20	0.85	0.21	10.80	4.23	8.74	1.89
	T/T	29	3.22	1.88	3.33	1.12	33.70	17.00	35.20	17.70	0.88	0.28	11.80	3.99	9.37	1.45
*UGT2B7* rs5013211	A/A	30	3.28	1.87	3.28	1.13	33.80	16.70	35.20	17.40	0.87	0.28	11.60	3.98	9.34	1.43
	G/A	28	2.97	1.33	2.87	0.86	31.20	11.30	32.10	11.40	0.86	0.21	10.90	4.27	8.75	1.92
	G/G	10	2.79	1.57	3.48	1.08	29.20	12.50	30.10	12.90	0.88	0.22	11.20	3.80	8.74	1.27
*UGT2B7* rs4257713	A/A	5	2.23	1.18	3.73	1.31	24.80	6.37	25.60	6.22	0.97	0.17	12.70	3.82	8.93	1.24
	A/G	26	3.16	1.43	2.90	0.88	32.80	12.40	33.70	12.60	0.81	0.21	10.20	3.97	8.69	1.82
	G/G	37	3.14	1.77	3.23	1.07	32.50	15.70	33.80	16.30	0.89	0.26	11.80	4.03	9.24	1.55
*PEAR1* rs77235035	A/A	7	3.05	0.91	2.71	1.23	24.60	5.73	25.20	5.86	0.94	0.19	11.20	2.27	8.29	0.89
	A/C	36	3.05	1.73	3.14	0.98	33.40	16.60	34.70	17.20	0.89	0.28	11.80	4.71	9.20	1.75
	C/C	25	3.13	1.63	3.26	1.05	32.10	10.90	33.10	10.90	0.81	0.18	10.50	3.29	8.93	1.62
*PEAR1* rs735953	C/C	8	3.14	1.87	2.75	1.12	29.00	19.80	30.10	21.00	0.96	0.25	12.30	3.90	8.93	1.51
	C/T	33	3.08	1.61	3.11	1.02	32.90	15.20	34.20	15.60	0.87	0.27	11.50	4.62	9.08	1.77
	T/T	27	3.06	1.59	3.30	1.02	31.80	10.70	32.90	10.70	0.83	0.19	10.70	3.30	8.94	1.55
*PEAR1* rs189160314	A/A	66	3.04	1.60	3.16	1.04	31.90	14.20	33.10	14.60	0.87	0.24	11.40	4.05	9.06	1.61
	G/A	2	4.48	1.88	2.50	0.24	34.80	10.90	35.20	10.60	0.78	0.05	7.97	1.64	7.18	1.90
*SLCO1B1* rs4149032	C/C	15	3.23	1.93	2.96	0.89	37.00	20.40	38.20	21.00	0.86	0.27	11.00	5.52	8.72	2.09
	C/T	37	2.91	1.30	3.25	1.07	29.80	9.27	30.80	9.62	0.89	0.23	11.50	3.58	8.93	1.57
	T/T	16	3.34	1.97	3.06	1.08	32.60	15.70	33.90	16.20	0.81	0.23	11.10	3.66	9.45	1.30
*SLCO1B1* rs4149034	A/A	15	3.23	1.99	3.13	1.08	32.40	16.20	33.80	16.70	0.82	0.23	11.30	3.68	9.51	1.32
	A/G	37	2.95	1.34	3.21	1.09	29.80	9.29	30.80	9.64	0.89	0.24	11.40	3.61	8.91	1.57
	G/G	16	3.25	1.86	2.99	0.87	36.80	19.70	38.00	20.30	0.85	0.26	10.90	5.34	8.75	2.02
*SLCO1B1* rs2306283	A/A	3	3.74	2.27	3.06	1.23	41.10	32.70	42.10	33.50	0.91	0.39	10.40	4.77	8.04	1.30
	A/G	31	2.98	1.55	3.26	1.03	32.40	13.50	33.80	13.90	0.91	0.26	12.30	4.70	9.35	1.77
	G/G	34	3.11	1.65	3.04	1.03	30.90	12.70	31.90	13.10	0.82	0.20	10.40	3.11	8.78	1.50
*SLCO1B1* rs4149057	C/C	4	3.03	2.33	3.04	1.00	34.60	29.60	35.70	30.20	0.97	0.35	13.00	6.45	9.10	2.37
	C/T	29	3.01	1.53	3.30	1.05	33.00	13.50	34.30	14.00	0.90	0.26	12.10	4.57	9.27	1.74
	T/T	35	3.14	1.64	3.02	1.02	30.90	12.50	31.90	12.90	0.82	0.20	10.40	3.07	8.78	1.48
*SLCO1B1* rs2291075	C/C	19	3.38	1.62	3.07	1.06	33.70	14.80	35.00	15.20	0.86	0.26	11.50	4.24	9.32	1.57
	T/C	31	2.96	1.49	3.30	1.00	32.10	13.30	33.30	13.70	0.87	0.24	11.40	4.36	9.10	1.75
	T/T	18	2.96	1.84	2.95	1.06	30.10	14.90	31.10	15.50	0.88	0.22	10.70	3.34	8.52	1.47
*SLCO1B1* rs2291076	C/C	35	3.14	1.64	3.02	1.02	30.90	12.50	31.90	12.90	0.82	0.20	10.40	3.07	8.78	1.48
	T/C	28	3.02	1.56	3.35	1.04	33.00	13.80	34.40	14.30	0.90	0.27	12.20	4.64	9.32	1.75
	T/T	5	2.95	2.02	2.83	0.99	34.30	25.70	35.30	26.20	0.95	0.30	12.40	5.73	8.88	2.10
*ABCB1* rs2235048	A/A	22	2.70	1.15	3.26	1.03	29.90	10.20	30.80	10.10	0.82	0.18	10.90	3.72	9.08	1.71
	G/A	35	3.21	1.70	3.15	1.04	32.80	15.30	34.00	15.90	0.91	0.27	11.80	4.40	9.01	1.71
	G/G	11	3.41	2.05	2.88	1.04	34.00	16.90	35.30	17.60	0.82	0.22	10.40	3.49	8.85	1.33
*ABCB1* rs2235047	A/A	23	3.32	1.95	3.01	1.01	33.30	16.20	34.60	16.90	0.86	0.23	11.30	4.41	9.02	1.68
	C/A	37	3.05	1.48	3.28	1.09	32.00	12.80	33.10	13.20	0.87	0.25	11.20	3.71	8.93	1.59
	C/C	8	2.54	1.08	2.90	0.78	28.70	13.60	29.60	13.50	0.86	0.22	11.80	4.87	9.31	1.94
*ABCB1* rs1045642	A/A	11	3.41	2.05	2.88	1.04	34.00	16.90	35.30	17.60	0.82	0.22	10.40	3.49	8.85	1.33
	A/G	35	3.21	1.70	3.15	1.04	32.80	15.30	34.00	15.90	0.91	0.27	11.80	4.40	9.01	1.71
	G/G	22	2.70	1.15	3.26	1.03	29.90	10.20	30.80	10.10	0.82	0.18	10.90	3.72	9.08	1.71
*ABCB1* rs2235013	C/C	34	3.22	1.80	3.12	1.05	33.50	17.10	34.70	17.60	0.87	0.27	11.40	4.41	9.06	1.59
	T/C	28	3.03	1.45	3.11	1.02	30.50	10.20	31.60	10.70	0.88	0.21	11.20	3.39	8.91	1.63
	T/T	6	2.56	1.20	3.44	1.07	31.00	10.50	32.00	10.10	0.83	0.21	11.30	5.26	9.15	2.20
*ABCB1* rs2235033	A/A	34	3.22	1.80	3.12	1.05	33.50	17.10	34.70	17.60	0.87	0.27	11.40	4.41	9.06	1.59
	G/A	28	3.03	1.45	3.11	1.02	30.50	10.20	31.60	10.70	0.88	0.21	11.20	3.39	8.91	1.63
	G/G	6	2.56	1.20	3.44	1.07	31.00	10.50	32.00	10.10	0.83	0.21	11.30	5.26	9.15	2.20

*C*_*max*_ */W*, maximum plasma concentration/weight ratio; *SD*, standard deviation; *T*_*max*_, time to reach maximum plasma concentration; *AUC*_*0–t*_ */W*, area under the curve between the predose and final time points/weight; *AUC*_*0–∞t*_ */W*, AUC between the predose and infinity/weight; *CL/F*, apparent drug clearance; *V*_*d*_ */F*, apparent volume of distribution; *T*_*1/2*_, half-life

## Discussion

Ticagrelor has been considered a new P2Y_12_ inhibitor and has reportedly overcome clopidogrel resistance. Although ticagrelor non-responders only account for 3% of patients with ACS, the pathological consequences can be devastating (Thomas and Storey, 2016; Laurent et al., 2021). Currently, the US Food and Drug Administration (FDA) reported that ticagrelor does not need dose adjustment for different races. However, owing to the East Asian paradox, East Asian patients with ACS are at a higher risk of major bleeding than ischemic risk compared to Caucasians; hence, a unique antiplatelet strategy needs to be urgently established for East Asian individuals on the basis of reliable clinical and experimental evidence (Kim et al., 2021). This study investigated the impact of gene polymorphisms on the PK of ticagrelor, which could facilitate personalized antiplatelet therapy in East Asian patients with ACS. To the best of our knowledge, this study included the largest number of SNPs that may have an effect on the PK of ticagrelor, as revealed through whole-exome sequencing among healthy Chinese volunteers. Our study is the first to show that *CYP4F2* rs2074900 was significantly associated with the PK of ticagrelor.

In this study, women were more likely to exhibit higher values of AUC/*W* and *C*_max_/*W* than men, a result similar to that previously reported for healthy volunteers (Teng et al., 2012). A possible explanation for the increased ticagrelor and AR-C124910XX exposure may be the gender-related physical difference. Since renal clearance plays a minor role in ticagrelor excretion, gender-related differences in renal function are unlikely to be associated with the PK of ticagrelor and AR-C124910XX (Teng et al., 2012). Another reason could be the gender-specific difference in CYP3A activity. Endogenous sex hormones reportedly affect CYP3A activity (Yoon et al., 2021). Ticagrelor is also a P-glycoprotein substrate. A lower P-glycoprotein activity in women than in men could result in a decreased biliary excretion of ticagrelor/AR-C124910XX, leading to a concomitant increase in the blood levels (Wang et al., 2021). However, dose adjustment is considered unnecessary on the basis of gender (Teng, 2015).

Furthermore, food showed considerable effects on the PK of ticagrelor in our study. On consuming a standard high-fat breakfast, the AUC/*W* values were found to be higher than those in the fasting condition, which was consistent with the FDA guidelines and with the results of a previous study (Wang et al., 2021). The FDA reported that a high-fat meal could result in a 21% increase in AUC. Nonetheless, the FDA suggests that ticagrelor can be taken with or without food, in accordance with the label. A possible explanation was that food had a minimal clinically significant effect, and this difference did not translate into changes in the pharmacodynamic (PD) profile of ticagrelor (Schilling et al., 2020).

Interestingly, we found that *CYP4F2* rs2074900 A/A carriers had higher *C*_max_/*W* and AUC/*W* values and lower CL/F and *V*_d_/F values than A/G and G/G carriers, implying that A/A carriers may experience a better therapeutic effect or unexpected bleeding events. *CYP4F2* belongs to the CYP4 subfamily of enzymes and is primarily expressed in the human liver and kidney. *CYP4F2* catalyzes the ω-hydroxylation of arachidonic acid to 20-HETE, which is a potent vasoconstrictor (Hirani et al., 2008; Zordoky and El-Kadi, 2010). In patients with ACS, *CYP4F2* polymorphisms have significantly affected antiplatelet therapy, and *CYP4F2* rs3093135 T/T carriers reportedly had a higher antiplatelet effect of ticagrelor than A/A and A/T carriers (Kupstyte et al., 2015; Tatarunas et al., 2020). The rs2074900 G>A mutation frequency is up to 22% in the East Asian population, as reported in the PharmGKB database (https://www.pharmgkb.org/page/dpwg), which is concurrent with our results; hence, it is worth further exploring whether *CYP4F2* rs2074900 polymorphisms impact the PK and PD of ticagrelor in patients with ACS.

Previous studies have suggested that *PEAR1* rs12041331 A/A homozygote carriers showed significantly increased inhibition of platelet aggregation (IPA) than did G allele carriers (Li et al., 2017b; Alhazzani et al., 2021). In general, there is a close affinity between the SNPs of *PEAR1* and platelet function. Furthermore, the *PEAR1* SNPs also influence the prognosis of patients with ACS (Stimpfle et al., 2018). The *PEAR1* rs77235035 C>A mutation frequency is 35.52% in the Asian population, according to the PharmGKB database, and this is the first study to report the effect of *PEAR1* variants on the PK of ticagrelor. In this study, the *PEAR1* rs77235035 A/A carriers exhibited lower AUC/*W* values than did A/C carriers, but not the C/C carriers. Thus, this finding could be spurious. We highly recommend verifying the association between *PEAR1* SNPs and ticagrelor in a large sample size.

*CYP3A4* and *CYP3A5* are the primary enzymes that convert ticagrelor to AC-R124910XX, the former playing a dominant role in the process. Three studies have reported the effect of *CYP3A* on the PK of ticagrelor in healthy volunteers. A previous study reported that CYP3A4*1G allele carriers had greater AR-C124910XX AUC_0–*t*_, AUC_0–∞_, and *C*_max_ and longer *T*_1/2_ than *1*1 carriers in healthy Chinese subjects (Liu et al., 2017). Nevertheless, another study reported that CYP3A4*1G was not related to the PK of ticagrelor and AR-C123410XX (Li et al., 2017a). Moreover, in Caucasian individuals, CYP3A4*22 carriers showed a higher exposure of ticagrelor than did non-carriers (Holmberg et al., 2019). In our study, the rs28371759 SNP was the only identified variant of *CYP3A4*. Nevertheless, the CYP3A4*18 (rs28371759) variant was not associated with ticagrelor and AR-C124910XX PK. Most studies primarily explored the potential interactions between ticagrelor and CYP3A4 inducers or inhibitors. Hence, further studies are required to investigate the effect of *CYP3A4*/*5* polymorphisms on the PK of ticagrelor.

*CYP2C19* is a key clopidogrel-metabolizing enzyme, and there is a strong association between CYP2P19*2 polymorphism and clopidogrel resistance in patients with coronary heart disease (CHD) (Sun et al., 2020). CYP2C19*2 and *CYP2C19*∗3 appeared to be the most common alleles in the Chinese population. A previous study reported that CYP2C19*3 G/G carriers had a higher *T*_max_ than G/A carriers after a single dose of ticagrelor (Zhu et al., 2019). In our study, CYP2C19*2 had no effect on the PK of ticagrelor. Ticagrelor is a putative P-glycoprotein substrate that could be encoded by *ABCB1*. As an efflux transporter, P-glycoprotein significantly affects drug disposition since it is expressed in intestinal mucosal epithelial cells, hepatocytes, and capillary endothelial cells (Zhu et al., 2019; Ji et al., 2021). It is reasonable to speculate that *ABCB1* polymorphisms are associated with the plasma exposure of ticagrelor. Nevertheless, concurrent with a previous study, the observed *ABCB1* variants, such as rs2235048, rs2235047, rs1045642, rs2235013, and rs2235033, were not associated with the PK of ticagrelor (Teng, 2015). In addition, the remaining *CYP2C9*, *CYP2B6*, *UGT2B7*, and *SLCO1B1* polymorphisms had no effect on the PK of ticagrelor and its metabolite AR-C124910XX, which is consistent with previous findings (Zhou et al., 2011; Zhu et al., 2019).

### Limitations

There are still some limitations in our study. Firstly, the PD of ticagrelor was not evaluated, especially platelet aggregation; hence, the identified SNPs that affected the PK of ticagrelor in this study remain to be further confirmed in patients with ACS. Secondly, the sample size was not large enough to identify all gene variants associated with the PK of ticagrelor. However, our study included the largest number of candidate SNPs for the analysis of their relationship with the PK of ticagrelor. In addition, as the bioequivalence study design excluded confounding factors, the results are relatively reliable.

## Conclusion

This study included the largest number of candidate SNPs in analyzing the association between gene variants and the PK of ticagrelor and AR-C124910XX using whole-exome sequencing. This study is the first to report that *CYP4F2* rs2074900 has a remarkable impact on ticagrelor PK variability. In addition, sex and food also impacted the PK profiles of ticagrelor and AR-C124910XX. Further studies are warranted to confirm our findings.

## Data Availability

The data presented in the study are deposited in the Sequences Read Archive database at the NCBI under accession number PRJNA798857.
