# Supplementary material for: Effect of CYP4F2 Polymorphisms on Ticagrelor Pharmacokinetics in Healthy Chinese Volunteers
Source: Front Pharmacol. 2022 Feb 25;12:797278. doi: 10.3389/fphar.2021.797278 (PMC8915292; doi:10.3389/fphar.2021.797278)
Supplement: Supplementary file 1 [file Table1.docx]

Supplement Table S1

Summary of accuracy, precision of ticagrelor and AR-C124910XX in human plasma by LC-MS/MS using ticagrelor-D7 and AR-C124910XX-D7 as the internal standard.

| Parameters | Conditions | LLOQ | LQC | GMQC | MQC | HQC |
| --- | --- | --- | --- | --- | --- | --- |
| Ticagrelor |  | 5ng/ml | 15ng/ml | 150ng/ml | 800ng/ml | 1600ng/ml |
| Accuracy (RE, %)^a^ | Intra-assay | 14 | 8.7 | 6.0 | 5.3 | 6.9 |
|  | Inter-assay | 13.4 | 7.3 | 2.7 | 3.5 | 2.5 |
| Precision (CV, %)^b^ | Intra-assay | 5.2 | 4.8 | 3.9 | 2.8 | 1.8 |
|  | Inter-assay | 4.3 | 4.5 | 3.5 | 2.6 | 3.7 |
| AR-C124910XX |  | 2.5ng/ml | 7.5ng/ml | 75ng/ml | 400ng/ml | 800ng/ml |
| Accuracy (RE, %)^a^ | Intra-assay | 13.2 | 9.2 | 4.5 | 7.3 | 9.6 |
|  | Inter-assay | 6.8 | 6.3 | 3.1 | 5.8 | 5.5 |
| Precision (CV, %)^b^ | Intra-assay | 11.5 | 5.5 | 4.0 | 1.9 | 2.6 |
|  | Inter-assay | 9.0 | 5.0 | 3.9 | 2.7 | 4.0 |

Notes: a expressed in percentage largest difference from the actual value in three batches; b expressed in percentage largest difference from the actual value in three batches; each concentration of QC had a sample size of N=6.

Abbreviations: LC-MS/MS, liquid chromatography-tandem mass spectrometry; QC, quality controls; LLOQ, lowest limit of quantitation; LQC, low-quality control; GMQC, geometric mean-quality control; MQC, middle-quality control; HQC, high-quality control; RE, relative error; CV, coefficient of variation.
